# MONTH-LONG FITNESS CHALLENGES IMPROVE ENDURANCE AND STRENGTH FOR VETERANS PARTICIPATING IN GEROFIT

**DOI:** 10.1093/geroni/igae098.3488

**Published:** 2024-12-31

**Authors:** Julie Rekant, Jamie Giffuni, Odessa Addison

**Affiliations:** Baltimore VA Medical Center, Baltimore, Maryland, United States; Baltimore and Loch Raven VAMC, Baltimore, Maryland, United States; University of Maryland School of Medicine, Baltimore, Maryland, United States

## Abstract

Older adults are vulnerable to loneliness and low physical activity engagement. Peer accountability can help encourage participation in exercise and foster a sense of community. Two, month-long fitness challenges were offered to older Veterans (65+ years) in Gerofit: (1) “The Long March” was a walking challenge with daily step goal targets increasing each week, (2) “Squatober” was a sit to stand challenge with daily bodyweight squat repetition goals increasing each week. Veterans were provided with cardio and strength workouts to complete in addition to the daily challenges. Thirty-one unique Veterans participated in these challenges (9 Veterans participated in both). Veterans in The Long March challenge (N=18, Age: 72.8±4.5 years, BMI: 30.2±5.9 kg/m2, 83% Male, 78% Black) participated in 88.3±28.0% of challenge days and met their step goals 50.2±36.1% of the days they participated. Veterans in The Long March challenge increased their Six Minute Walk Test distances by 28.0±48.7 meters after four weeks. Twenty-two Veterans participated in the Squatober challenge (Age: 74.3±6.3 years, BMI: 30.7±5.2 kg/m2, 81% Male, 86% Black). Sixty-three percent (N=14/22) completed every workout. Veterans participating in the Squatober challenge reported an overall rating of perceived exertion of 5.0±2.5 out of 10, decreased their Five Time Sit to Stand times by 1.3±2.3 seconds, and were able to complete an average of 2.6±4.1 more sit to stands during the 30 Second Chair Stand Test after four weeks. Older Veterans found fitness challenges engaging and showed functional improvements in just four weeks with daily movement goals.

